# Pleuro-Pneumonitis Complicated with Pericarditis, Masked by Delirium

**Published:** 1850-02

**Authors:** Austin Flint

**Affiliations:** One of the attending physicians to the Buffalo Hospital of the Sisters of Charity. Buffalo


BUFFALO MEDICAL JOURNAL

AND

MONTHLY REVIEW.

VOL. 5. FEBRUARY, 1850. NO. 9

### ORIGINAL COMMUNICATIONS.

ART. I.—*Pleuro-pneumonitis complicated with Pericarditis, masked by delirium.* By AUSTIN FLINT, M. D., one of the attending physicians to the Buffalo Hospital of the Sisters of Charity.

Philip Crotty, apparently about 35 years of age, was brought to the Hospital on the 10th of December, 1849, by two men, who seemed to be intoxicated, and nothing was ascertained relative to the previous history of the case.

He was actively delirious during the night, constantly attempting to get out of bed, and muttering. He appeared to have visual illusions. The attendants thought he was intoxicated.

On the 11th Dec., A. M., he was tranquil, and when spoken to made no reply, shaking his head. Eyes had a wild, staring expression. Face presented cutaneous congestion. Teeth encrusted with sordes. He could not be made to protrude his tongue, but, in so far as it could be examined by depressing the lower jaw, it was thinly coated, and dry. Abdomen retracted. No eruption on chest, or abdomen. No abdominal tenderness. He had urinated in bed during the night. Had not asked for food or drink. Skin warm and moist. Upper extremeties presented cutaneous capillary congestion. Pulse small, feeble, and not accelerated. Some picking of bed clothes; hand most of the time during the examination of the case was working at one of the buttons of his shirt.

*Treatment:*—Let the hair be cut close to the head. If active delirium recur, give tart. ant. et pot., gr. 1-8, hourly, omitting if it occasion nausea. Mustard pediluvia. Cold applications to the head.

12th, A. M.—Passed the day, yesterday, quietly; exhibited delirium at evening, but after taking the antimony became quiet and so continued during the night. Appearing prostrated, a little brandy was given. Now lies awake, but keeps his eyes fixed on the bed. Cheeks present deep capillary congestion. Will not protrude his tongue, nor reply to questions. Is docile; makes no resistance to examinations, or moving the body. Tongue, so far as can be examined, is moist. Abdomen flat, soft, without tenderness. No eruption. Has had no dejection. Passes urine in bed. Muttered somewhat during night. Asks for nothing, and drinks, as well as medicine, are forcibly administered.

Emp. vesicat., 8 by $2\frac{1}{2}$, to nucha. Repeat tart. ant. et pot. if febrile movement or delirium occur. Mustard pediluvium night and morning. Cold applications to head.

13th.—Aspect somewhat improved. Talkative during night, but did not attempt to get out of bed. Urinates in bed. Asks for nothing. Blister has vesicated well, Lies wakeful most of the day, with his eyes fixed on one object. Changes his position occasionally. No dejection. Took milk porridge twice during night.

Tart. ant. et pot., grs. 1-8, every two hours, omitting if it nauseates or prostrates. He has had brandy occasionally. Let this be omitted unless there are evidences of sinking.

14th.—Delirious during greater part of night, talking to himself, manœuvering with the bed clothes, and picking at invisible objects. Became quiet subsequently, and this morning asked for food. Said he would like some toast and tea. He is quite deaf. Replies to questions. Says he is better, but thinks he has committed some crime, and says he has been out of his senses. His mind evidently ladors under some delusions. Aspect improved. Protrudes his tongue readily. It is clean, and dry in the centre. Face and upper extremities continue congested. Skin cool. Pulse 92, soft. He was got up last evening, and passed fœces and urine. Did not intimate any desire for these evacuations, but was got up for that purpose. Has not urinated in bed since yesterday morning. Used the urinal this morning. Abdomen not distended. No eruption. The antimony was continued through the day, yesterday, up to nine last evening. It did not vomit him, but he seemed to be slightly nauseated. Has had no perspiration. Got about half an ounce of brandy every four hours yesterday. Medicine, drink, and nutriment were given, yesterday, with a good deal of trouble, owing to his resistance. Continue brandy, half an ounce, every four hours, and nutritious diet.

15th.—Between nine and ten, P. M., last evening, he became actively delirious—shouting, and crying, as if from apprehension of danger. Continued so until 4, A. M., of to-day, and since then has been quiet. Ate toast and tea for breakfast, and seemed rational. Now sleeping, and, lest I should awaken him, I did not prosecute examination of symptoms.

16th.—Passed a quiet night; seemed rational this morning. Took food with relish. Says he is better. Pulse (not enumerated) moderately accelerated, small, and soft. Skin warm and moist.

18th.—Actively delirious night before last; more quiet last night. Labors under the delusion of danger from personal violence. Countenance presents a fixed, and somewhat frowning expression (expressive of a mental state, not sensibility to light). Protrudes his tongue, which is red, dry, and scabby. Had copious dejection yesterday, natural in appearance. Gets up to urinate. Urine deposits lateritious sediment. Skin warm. Pulse somewhat accelerated, soft. Respiration normal.

Let his head be shaved, and apply emp. vesicat., 6 by 6. Rub into axillary and inguinal regions, successively, ung. hydrarg. ℥ss. night and morning.

19th. Passed a quiet night. Reports better. Aspect improved. Slight frowning continues. Tongue thinly coated and dry. No dejection. Skin warm and dry. Pulse 100, soft, compressible. Asks for water frequently, but has no desire for food. Mind appears to be dull—difficulty of apprehending the import of questions. When asked repeatedly if he has pain, and where, he replies in the affirmative, and places his hand on the occiput. No distension of the abdomen. No tenderness. No eruption. Blister to scalp not applied until this morning. Made no resistance to shaving the head. Cont. inunction.

22d. Active delirium on the night of the 19th, so that it was necessary to transfer him from the general ward to a room by himself. Has been actively delirious succeeding nights until last night. Quiet through the day. Now lying quietly. When asked how he is, he replies, "*guilty.*" This answer he returned to the question several times repeated. Lies with his eyes fixed upon the bed. Takes no observation of persons or things around him. Does not look up when spoken to. Protrudes his tongue when desired. It is reddened, clean, and inclined to dryness. Skin rather hot and dry. Pulse 120, soft. Bowels soluble. No vomiting. Blister on cranium vesicated moderately. Inunction has been continued, but sometimes imperfectly performed, owing to his resistance.

Iodid. potassii, grs. x., three times daily. Cont. inunction.

23d. Talkative during night, but did not attempt to get out of bed. Passed urine in bed, but fœces in stool, having been got up for that purpose. His muttering denotes that he thinks he is in jail. Now sleeping, but roused, without difficulty, and with a sudden start. Says, on being asked how he is, that he "is very well, and as strong as a horse." Face congested. Sordes on teeth. Respiration is, and has been normal. No cough. Tongue clean but dry. Dejection this morning large solid and natural. Skin warm and dry. Pulse 112, feeble. Took some food.

24th. This morning, at about two o'clock, complained of pain in the inferior lateral portion of left chest. Continued to complain for several hours. Sleeping on my approaching his bedside, but readily roused, and referred me to the seat of the pain. Said it was a sharp, catching pain. He has no cough, nor expectoration.

On percussion of inferior anterior and lateral portion of left chest, tympanitic resonance, evidently denoting presence of gas in stomach, not sufficiently intense to indicate pneumothorax. No respiratory sounds. Posteriorly, flatness over the left inferior portion of chest. Slight crepitant rale in the latter region at end of inspiration. He seems rational. Teeth covered with sordes. Tongue dry and hard. Skin warm and dry. Pulse 128, small. Dejection yesterday. Gums look reddened and swelled, but he says his mouth is not sore. No mercurial fœtor. Inspiration somewhat shortened and quickened. No dilation of *alae nasi.* Respirations 24.

Discontinue mercurial inunction, and the iodid. potassii. S. Quiniae, grs. i., pulv. Doveri, grs. iii., three times. Carb. ammoniæ, grs. iv., every four hours. Milk and farinaceous diet.

25th. Several dejections during the night, none passed in bed. Asked for the vessel. This morning awake. Cannot be made to answer questions or protrude his tongue. Wishes to know why he was not hung before, etc. Skin warm. Pulse too indistinct to be enumerated. Continued treatment.

26th. Passed a quiet night. Now awake. Exhibits tremor of upper and lower extremeties, (possibly from cold). Moans with respiration. Pulse 120, so feeble and small as with difficulty to be enumerated. Complains of pain in left shoulder, and beneath ribs of left side. Tongue readily protruded, dry, fissured. No cough nor expectoration. Replies to questions coherently, but continues talking, and with incoherence. Disposed to be talkative. Says his pain in the side is a stitch.

Flatness on percussion over left chest posteriorly. Tympanitic resonance anteriorly and laterally, tubular respiration posteriorly, and bronchophony. Continued treatment, adding brandy, ℥ss every four hours.

27th. Sleeping quietly, but easily roused. Looked at me and smiled. This is the first time he has been observed to look pleasantly. Five or six dejections during night. Complained of pain beneath ribs of left side. When asked how he feels, said, "sometimes good, and sometimes bad." Teeth covered with sordes. Tongue dry and hard. When a question is addressed to him, he commences talking in an incoherent manner, and is not disposed to stop. Pulse filiform, two pulsations occur in quick succession, followed by an intermission of several beats. Skin warm and dry. Respirations, 32. Inspiration rather quick. Dilation of alae nasi. He seems in excellent spirits; asked me, when leaving his bedside, if I "was not going to bid him good bye," and laughed aloud.

Continue treatment, giving enema of t. opii., if dejections are frequent.

Death occurred the night of this day, preceded by coma for several hours.

*Autopsy about twelve hours after death.*—Head was first examined. The superficial veins of the brain presented considerable congestion. Aracknoid membrane over the superior surface of cerebrum, exhibited slight opacity. No effusion of fibrin otherwise appreciable. No sub-arachnoid serous infiltration. Cut surfaces of cerebral substance presented red points. Ventricles empty. Choroid pexus moderately congested. Consistence of cerebral substance normal. Effusion of serum, slightly turbid, in the arachnoid cavity, the quantity collected at the base of the cranium, and escaping from the spinal cord on elevating the body, amounting, by estimation, to between two and three ounces.

*Chest* was next examined. Right lung free from adhesions, and healthy. Usual amount of hypostatic congestion of dependent portion. Left pleural cavity contained, by estimation, about twelve ounces of turbid serum. Left lung, and costal pleura, presented large patches covered with fibrin, from three to five lines in thickness; in some portions semi-fluid, in other portions semi-organized. No adhesions, except nigh to the sternum. Both lobes of left lung solidified, not crepitating on pressure, and cut surfaces exuding serous fluid, but slightly spumous. No purulent infiltration, or abscesses.

*Heart.* Pericardium presenting universal layer of lymph, having the appearance of rugae, and several cords extending from the surface of the heart, to the pericardial sac, an inch, or more, in length. Large fibrinous coagula in each ventricle, without red corpuscles, not firmly adherent to endocardium Endocardial membrane normal. Valves sound.

Abdominal organs not examined, excepting the lower portion of the ileum, the lining membrane of which presented no morbid appearances.

*Remarks.*—The reader, having carefully perused the foregoing case, which is copied from the Book of Hospital Records, will, we imagine, have experienced some surprise on comparing the symptoms during life, with the appearances found after death. We are free to confess that the encephalic lesions were far less than we had expected to find, and that the views we had entertained of the disease, prior to the death of the patient, were falsified. Being uninformed concerning the history of the patient before his entering the hospital, we were unable to arrive at a positive diagnosis. At first we supposed it might be a case of fever, although the character of the delirium was different from that which usually attends the febrile career. As the case advanced, this supposition was found not to be tenable. We entertained some suspicions that it might prove to be a case illustrating a variety of *delirium ebriosum.* But the phenomena of its progress disproved, also, this idea. The symptoms seemed to us to denote that the head was chiefly and primarily affected, but whether the patient was laboring under subacute inflammation succeeding an acute attack, or under subacute inflammation developed *de novo,* we felt unable to decide. We are free to confess that we did not suspect the existence of pulmonary disease prior to his complaining of pain in the chest on the 24th. There were no rational indications of pulmonary disease—no cough, no expectoration, no embarrassment of respiration, no indications of pain, and, at first, little or no acceleration of the pulse. Nevertheless, overlooking the pulmonary affection was censurable, for physical exploration of the chest, at a previous date, would have disclosed the fact. The case may serve to exemplify the importance of physical exploration in cases in which there are no rational symptoms pointing to disease of the organs contained therein. That an earlier discovery of the pleuro-pneumonitis would probably been of little or no practical advantage to the patient, in so far as medical treatment is concerned, while it affords no apology, may serve to diminish the self-reproaches of the reporter. The non-discovery of the pericarditis is certainly far more excusable, inasmuch as the difficulty of diagnosis is vastly greater, and, under the circumstances, probably could not have been made with positiveness.

We would impress upon the attention of the reader the interesting illustration which this case affords of the existence of pleuro-pneumonitis, the entire left lung being withdrawn from the function of hæmatosis—the pleuritis unusually predominant, as a complication of pneumonitis—while not a single symptom existed suggesting the inquiry whether disease existed within the chest.

Having, at length, ascertained the existence of pneumonic inflammation, a conclusion, both natural, and, under the circumstances rational, was, that the encephalic disease had precedence, the lungs becoming subsequently affected, its development and progress being masked by the former. This succession of diseases it is known occasionally obtains. In view of the pathological appearances, however, that conclusion was probably erroneous. The inflammation of the pulmonic and cardiac structures, doubtless, first occurred, the head symptoms being secondary and dependent, but serving equally to mask the existence of the thoracic affections.

The practical lessons which this case enforces, then, are, first, that, in connection with extensive disease within the chest, cerebral symptoms may become developed, denoting that the head is the prime seat of disease, not only predominating over, but completely masking symptoms of thoracic inflammation, the morbid appearances which the brain presents being inadequate of themselves to explain the amount of its functional disturbance; and, second, that, in cases of this description, physical exploration of the chest is never to be omitted because symptoms of pulmonary disease are absent.

The existence of pericarditis simulating encephalitis, although overlooked by most systematic authors, has excited the attention of some observers. The reader will find some highly interesting and valuable remarks on this subject by Dr. Watson, in his published lectures [Am. edition, page 690, et seq]. Dr. Watson gives an account of several cases in which pericarditis was attended by delirium, inducing the belief that the brain was the chief, and only seat of mischief. Some of these cases occurred under his own observation; others are gathered from published reports by several writers. As Dr. Watson's work is in the hands of most practitioners, we shall content ourselves with thus merely referring to it. In all the cases presented by this author, the only thoracic disease existing was situated in the heart, and the latter had occurred as a complication of acute rheumatism. The case we have reported differs in the co-existence of pleuro-pneumonitis, and in the absence of any evidences of rheumatic disease. Dr. Watson points out one peculiarity in the delirium attendant on his cases, viz. it was characterized by taciturnity, and, when active, by unhappiness and apprehension. These peculiarities, the reader will not fail to note, mark the case we have given, until the last day of the patient's life, when he exhibited hilarity, Dr. W. also directs attention to the occurrence of deceptive symptoms of amendment. In this particular the analogy with the case we have reported holds good. The patient became more rational, and we entertained hopes, at times, of a favorable issue. We may remark here, that the occurrence of pain on the 24th, was doubtless owing to an improved condition of the brain in consequence of which the perception of painful sensations was experienced for the first time after the case came under our observation.

We find this subject more fully treated of in a late work on "*Disorders of the Cerebral Circulation,*" *by George Burrows, M. D.* As this work may be less accessible to the reader than the lectures by Dr. Watson, we will make some quotations, which, if we mistake not, the interest and importance of the subject will fully justify.

In commencing a chapter on "*Affections of the Brain and Spinal Cord depending on acute disease of Heart,*" [page 177] Dr. Burrows remarks:—

"Different systematic writers on diseases of the heart have incidentally mentioned, that inflammatory affections of that organ are sometimes accompanied with such severe symptoms of nervous irritation, that the primary affection of the heart is either overlooked altogether, or is so masked by the nervous disorder that it is not detected until irreparable mischief is done to a vital organ. A few cases of this kind are to be found recorded in periodicals, and in the Transactions of different medical societies, during the past twenty years. But a connected view of these remarkable and fearful cases has not, as far as I know, been hitherto presented to the medical public. Without arrogating to myself any merit for originality in the view of them which I am about to offer, I think a synopsis of them will be a suitable illustration of the great pathological principle I have been upholding in the previous sections, vix. the influence of modifications of the circulation on the functions of the brain.

The earliest recorded case of this kind is that detailed by Mr. Stanley.* Dr. Abercombie, in 1821, communicated a nearly similar case to the Medico-Chirurgical Society of Edinburgh, in a paper entitled, "Contributions to the Pathology of the Heart. It is singular that this valuable essay from so distinguished a physician should have escaped the notice of (I believe) all subsequent writers on diseases of the heart. Dr. Latham was the next in the order of precedence to call attention to this deceptive form of cardiac inflammation; and he informs us,† "that when he first related the particulars of his case to several medical friends, they looked incredulous, or rather contemptuous, of the man who would mistake an inflammation of the pericardium and heart for an inflammation of the brain." Nevertheless, I shall give a short account of many analogous cases, occurring in

* Transactions of the Medico-Chirurgical Society of London, vol. vii, 1817.

† Pathological Lectures on the Heart, London Medical Gazette, vol. iii.

the practice of men of eminence both in Paris and London. How many others have occurred in the practice of physicians who have been less candid in recording their mistakes, and how great a number must have happened in the practice of those who were unable, or who took no pains to distinguish these deceptive cases, it is impossible to say. Andral* and Bouillaud† have recorded cases of this kind, as well as Dr. Copland,‡ Dr. Macleod,§ Dr. F. Hawkins,|| and a few others in this country. But the most interesting and valuable information upon this subject has been given to the profession by Dr. Richard Bright, in his account of "Cases of Spasmodic disease accompanying Affections of the Pericardium."¶

From the histories of several cases, we select the following, as bearing the closest analogy to the case we have given:—

*Cases IV. and V.—Pericarditis, without any signs of Rheumatism, giving rise to Symptoms of Inflammation of the Brain.*

Dr. Latham has recorded** the case of a young woman, which is strongly impressed upon my recollection, who was admitted into St. Bartholomew's Hospital in 1828, and in whom all the symptoms led to the belief that the brain was inflamed. The whole force of the treatment was therefore directed to that organ. The woman died; and upon dissection, the brain and its coverings were found in a perfectly healthy and natural state, and the pericardium, towards which there was no symptom during life to induce the slightest suspicion of disease, exhibited unequivocal marks of acute inflammation.

*Case V.*—Another woman, æt. 40, was admitted, in 1839, into St. Bartholomew's Hospital, suffering under slight delirium, fever, and other symptoms of an inflammatory affection of the brain. She was treated for this supposed affection of the brain, and did not present a single symptom referable to the heart. She sank in about four days after admission. No disease was found in the brain or its membranes; the free surfaces of the pericardium were coated with thick honeycomb lymph, which had evidently been effused within a few days previous to her death.

There appears to be one peculiarity common to three out of the five cases I have just recited, which was, that, throughout their progress, there was no sympton present which directed attention to the organ affected. In the case recorded by Dr. Abercombie, the patient had, it is true, complained of pain in the epigastrium, and of dyspnœa, before the accession of the symptoms of affection of the brain and spinal cord; and these symptoms caused his treatment to be addressed near to the organ affected. Nevertheless, this case, as well as the other four, proved fatal.

In conclusion, with respect to the pathology of the affection of the brain giving rise to the cerebral symptoms in this case, we would say, briefly, that much is doubtless to be attributed to the disturbed circulation incident

---

* Clin. Med.

† Traite sur les Maladies du Cœur.

‡ Dictionary of Medicine.

§ On Rheumatism.

|| Gulstonian Lectures on Rheumatism.

¶ Med. Chir. Trans. vol. xxii.

** Medical Gazette, vol. iii.

to the pneumonic and cardiac lesions. The authors to whom we have referred, attribute the cerebral symptoms in the instances which they present, chiefly to this source. But, in addition to this, in the case of Crotty, there existed a slight degree of meningitis, denoted by the opacity of the arachnoid existing at the summit of the cerebral lobes. This would be sufficient, probably, to account for the activity of the delirium, and the situation of the meningeal inflammation is to be considered in connection with the character of the mental aberrations. The effusion into the arachnoid cavity, also, probably possesses considerable pathological importance in the case. The same amount of effusion occurring rapidly, judging from analogy with other cases that have come under our observation, by interrupting the functions of the medulla oblongata, and destroying the instinctive want of respiration, would have occasioned sudden death by apnœa. The effusion, probably, took place gradually. The phenomena of the case, in so far as they concern the brain, we regard as due to disturbed cerebral circulation, slight meningitis, and effusion, combined. It would be difficult to assign to each of these elements, its exact relative value. Altogether they induced a morbid condition of the encephalon sufficient to mask the combined thoracic diseases, the latter sustaining to that condition the relation of priority, and, in part, the relation of causation.

Buffalo, January, 1850.

